# Multicenter prospective clinical study to evaluate the prediction of short-term outcome in pregnant women with suspected preeclampsia (PROGNOSIS): study protocol

**DOI:** 10.1186/1471-2393-14-324

**Published:** 2014-09-18

**Authors:** Martin Hund, Deirdre Allegranza, Maria Schoedl, Peter Dilba, Wilma Verhagen-Kamerbeek, Holger Stepan

**Affiliations:** Roche Diagnostics International Ltd, Forrenstrasse 2, CH-6343 Rotkreuz, Switzerland; Roche Diagnostics GmbH, Penzberg, Germany; Department of Obstetrics, University of Leipzig, Leipzig, Germany

**Keywords:** Preeclampsia, HELLP syndrome, Eclampsia, Predictive markers, Angiogenic factors, Antiangiogenic factors, sFlt-1, PlGF, Maternal outcome, Neonatal outcome

## Abstract

**Background:**

Preeclampsia is defined as new onset of hypertension and proteinuria at gestational week 20 or after. However, use of these measures to predict preeclampsia before its clinical onset is unreliable, and evidence suggests that preeclampsia, eclampsia, or hemolysis, elevated liver enzymes and low platelet count (HELLP) syndrome may develop without hypertension or proteinuria being evident. Because of its unpredictability, varying clinical presentation and potential adverse outcomes, pregnant women with suspected preeclampsia require intensive monitoring or hospitalization. Beyond preeclampsia diagnosis, there is a high unmet medical need for more reliable predictive markers for preeclampsia to improve maternal and fetal outcomes and reduce unnecessary hospital admissions. An imbalance of circulating angiogenic and antiangiogenic factors, including raised soluble fms-like tyrosine kinase-1 (sFlt-1) and decreased placental growth factor (PlGF), has been found in women diagnosed with preeclampsia and before clinical onset of the disease. The PRediction of short-term Outcome in preGNant wOmen with Suspected preeclampsIa Study (PROGNOSIS) was designed to investigate the use of the sFlt-1/PlGF ratio in the short-term prediction of preeclampsia.

**Methods/Design:**

This global, multicenter, prospective, double-blind, non-interventional study aims to derive and validate cutoffs for the sFlt-1/PlGF ratio, to rule out (for 1 week) or rule in (within 4 weeks) the occurrence of preeclampsia/eclampsia/HELLP syndrome. Eligible participants are women presenting at 24 to <37 weeks’ gestation with clinical suspicion of, but not manifest preeclampsia/eclampsia/HELLP syndrome. Clinical assessments, maternal serum sFlt-1/PlGF sampling and documentation of maternal/neonatal outcomes are performed at regular intervals, using strict diagnostic criteria for preeclampsia-related conditions and outcomes. Serum sFlt-1 and PlGF analysis will be performed using fully automated Elecsys® immunoassays. Investigators and participants will remain blinded to the results. Target recruitment is 1000 participants. Health economic analysis is also planned.

**Discussion:**

The results of PROGNOSIS will provide the most comprehensive evidence to date on the accuracy of the sFlt-1/PlGF ratio for short-term prediction of preeclampsia/eclampsia/HELLP syndrome. Adoption of the sFlt-1/PlGF test in clinical practice has the potential to reduce the frequency of adverse pregnancy outcomes for both mother and fetus, and decrease healthcare costs associated with unnecessary hospitalization of women with suspected preeclampsia.

## Background

Preeclampsia is a serious multi-organ complication in pregnant women defined by the new onset of hypertension and proteinuria at gestational week 20 or after [1–3]. It is a leading cause of fetal and maternal morbidity and mortality [2, 4, 5] and represents a considerable healthcare resources burden in developed countries.

The current "gold standard" for preeclampsia diagnosis involves blood pressure measurement and determination of protein in urine. However, because of its syndromal nature and the varying clinical presentation of preeclampsia phenotypes, the specificity and reliability of these assessments to predict who will develop preeclampsia, eclampsia, or hemolysis, elevated liver enzymes and low platelet count (HELLP) syndrome is poor [6]. As a consequence, women with signs or symptoms associated with preeclampsia are often unnecessarily hospitalized for intensive monitoring until preeclampsia is ruled out. Conversely, women who require hospitalization may be overlooked because preeclampsia was not predicted based on the current diagnostic criteria. Improving the sensitivity and accuracy of assays for predicting preeclampsia has the potential to prevent over-diagnosis and over-treatment of women with suspected preeclampsia and may allow more efficient allocation of healthcare resources according to the patient’s risk [7].

It is estimated that one-fifth of antenatal admissions, two-thirds of referrals to day-care assessment units and one-quarter of obstetric admissions to intensive care units in developed countries are preeclampsia-related [8]. Healthcare costs for preeclampsia are high due to a high rate of cesarean deliveries, premature births and an increased requirement for neonatal care [4, 9]. World Health Organization figures indicate that hypertension during pregnancy accounts for 16% of maternal deaths in industrialized countries [10] and up to 25% in developing countries [4], even though most deaths due to preeclampsia and eclampsia are avoidable through timely diagnosis and management.

Although the etiology is not yet completely understood, preeclampsia is a heterogeneous syndrome driven by disturbed placental function in early pregnancy and an imbalance of angiogenic factors, such as soluble fms-like tyrosine kinase-1 (sFlt-1; also known as sVEGFR-1), placental growth factor (PlGF) and soluble endoglin (sEng). In preeclampsia, excess placental secretion of sFlt-1 and sEng inhibits vascular endothelial growth factor (VEGF) and transforming growth factor β1 signaling, respectively, resulting in endothelial cell dysfunction. sFlt-1 also antagonizes circulating pro-angiogenic PlGF, resulting in decreased PlGF expression in preeclamptic women [11]. Late-onset preeclampsia (after 34 weeks) is associated with less dramatic dysregulation of angiogenic factors than early-onset preeclampsia [12].

The ratio between the anti-angiogenic factor sFlt-1 and pro-angiogenic PlGF has been shown to be elevated in women with diagnosed preeclampsia and markedly elevated before clinical onset [11, 13–16]. Recent data from a case-control study defined separate cutoffs in early-onset and late-onset preeclampsia based on the Elecsys® sFlt-1/PlGF ratio. In early-onset preeclampsia, between 20 + 0 and 33 + 6 weeks of gestation, an sFlt-1/PlGF ratio of ≤33 was negative, and an sFlt-1/PlGF ratio of ≥85 was positive for confirmation of preeclampsia or HELLP syndrome, with a sensitivity/specificity of 95%/94% and 88%/99%, respectively. In late-onset preeclampsia developed after 34 + 0 weeks of gestation, an sFlt-1/PlGF ratio below the cutoff of ≤33 was negative, and an sFlt-1/PlGF ratio of ≥110 was positive. The cutoffs in late-onset preeclampsia have lower sensitivity/specificity (90%/73% and 58%/96%, respectively) than the cutoffs for early-onset preeclampsia [17].

Reliable and reproducible biomarker tests are important when considering the predictive value of sFlt-1, PlGF and the sFlt-1/PlGF ratio. Elecsys® sFlt-1 and Elecsys® PlGF (Roche Diagnostics GmbH, Mannheim, Germany) are the first fully automated immunoassays for the detection of preeclampsia biomarkers in maternal serum, and were used in the studies to determine clinically useful cutoffs. The assays provide convenient, rapid and reliable quantification of sFlt-1 and PlGF, and are currently CE-IVD approved for use as an aid in the diagnosis of preeclampsia in conjunction with other clinical findings [17–19]. Beyond diagnosis, however, there remains a high unmet medical need for reliable short-term prediction of preeclampsia in pregnant women with suspected preeclampsia. Addressing this unmet need may reduce the cost of monitoring by reducing unnecessary hospital admissions, and improve maternal and perinatal outcomes through earlier and better targeted management [7]. The PRediction of short-term Outcome in preGNant wOmen with Suspected preeclampsIa Study (PROGNOSIS) was designed to demonstrate the utility of the sFlt-1/PlGF ratio, as determined by the Elecsys® sFlt-1 and Elecsys® PlGF assays, in the short-term (up to 4 weeks) prediction of preeclampsia.

## Methods/Design

### Objectives

#### Primary study objectives

The primary objectives are:

1. To demonstrate that low ratios of sFlt-1/PlGF predict absence of preeclampsia/eclampsia/HELLP syndrome for 1 week after the baseline visit ("rule-out").
2. To demonstrate that high ratios of sFlt-1/PlGF predict diagnosis of preeclampsia/eclampsia/HELLP syndrome within 4 weeks after the baseline visit ("rule-in").

Cutoff-based algorithms will be developed as prediction models for each of these short-term outcomes using the serum sFlt-1/PlGF ratio. Should gestational age enhance the prediction performance, it will also be included.

#### Secondary study objectives

All secondary objectives will be analyzed in an exploratory manner. Analyses will be performed to investigate the utility of the sFlt-1/PlGF ratio in the short-term prediction of preeclampsia-related adverse outcomes. Objectives are:

-  To collect evidence that low ratios of sFlt-1/PlGF correlate with absence (within 1 week of baseline visit), and high ratios correlation with presence (within 4 weeks), of maternal preeclampsia-related adverse outcomes (other than preeclampsia/eclampsia/HELLP syndrome, which is included in the primary objective) or fetal preeclampsia-related adverse outcomes.

Maternal preeclampsia-related adverse outcomes are defined as maternal death, pulmonary edema, acute renal failure, cerebral hemorrhage, and cerebral thrombosis or disseminated intravascular coagulation. Fetal preeclampsia-related adverse outcomes are defined as perinatal/fetal death, iatrogenic delivery earlier than 34 weeks, intrauterine growth restriction (IUGR), placental abruption, respiratory distress, necrotizing enterocolitis or intraventricular hemorrhage.

Participants who develop preeclampsia/eclampsia/HELLP syndrome will be compared with gestational age-matched controls in a nested case-control collective to evaluate the performance of the sFlt-1/PlGF ratio, sFlt-1 and PlGF as an aid in diagnosis.

Other secondary objectives are to explore correlations between:

-  weekly increase of ratios of sFlt-1/PlGF and diagnosis of preeclampsia/eclampsia/HELLP syndrome within 4 weeks after the baseline visit.
-  preeclampsia severity, sFlt-1/PlGF ratio and sFlt-1 and PlGF levels at the time of preeclampsia diagnosis.
-  preeclampsia severity, changes in sFlt-1/PlGF ratio and sFlt-1 and PlGF levels during the week before diagnosis of preeclampsia/eclampsia/HELLP syndrome.
-  high ratios of sFlt-1/PlGF and preterm delivery.
-  ratios of sFlt-1/PlGF and time to delivery.

Another secondary objective is to investigate the potential economic benefit of the sFlt-1/PlGF ratio for informing decisions on the length of hospitalization of women with clinical suspicion of preeclampsia and newborns.

### Study design

PROGNOSIS is a multicenter, prospective, double-blind, non-interventional study designed to derive (part 1) and to validate (part 2) an sFlt-1/PlGF ratio cutoff-based short-term prediction model for each of the two scenarios described in the primary objectives:

1. To rule out preeclampsia/eclampsia/HELLP syndrome occurring within 1 week of assessment.
2. To predict that the participant will progress to develop preeclampsia/eclampsia/HELLP syndrome within 4 weeks of assessment.

Double-blinding is achieved by having study samples stored and analyzed at an independent analytical laboratory until after completion of parts 1 and 2. This approach ensures that neither the investigator nor the subject will have access to the results of the biomarker assessments. In this way, the sFlt-1/PlGF ratio remains unknown both to the investigator and to the study participant during the study.

### Target population

#### Inclusion criteria

Eligible participants are pregnant women aged 18 years or over, at a gestational age between week 24 + 0 days and week 36 + 6 days at the time of the first (baseline) visit. All participants are to have suspected preeclampsia diagnosed clinically per the protocol-defined criteria (Table 1) to ensure consistency across all sites. The percentage of women with suspected preeclampsia due to abnormal uterine perfusion is not to exceed 25% at any individual clinical site, and no more than 50% of enrolled participants at each site are to be beyond gestational week 32 + 0 at the baseline visit.

**Table 1:** Criteria contributing to suspicion of clinical diagnosis of preeclampsia*

Clinical signs and symptoms	
a. New onset of elevated blood pressure^a^	
b. Aggravation of pre-existing hypertension	
c. New onset of protein in urine^b^	
d. Aggravation of pre-existing proteinuria	
e. One or more other reason(s) for clinical suspicion of preeclampsia (see i. and ii.)	
**i. Preeclampsia-related symptoms:**	1. Epigastric pain
	2. Excessive edema/severe swelling, (face, hands, feet)
	3. Headache
	4. Visual disturbances
	5. Sudden weight gain (>1 kg/week in the third trimester)
**ii. Preeclampsia-relatedfindings:**	1. Low platelets
	2. Elevated liver transaminases
	3. (Suspected) intrauterine growth restriction
	4. Abnormal uterine perfusion detected by Doppler sonography with mean pulsatility index >95th percentile in the second trimester and/or bilateral uterine artery notching

^a^Does not need to be defined hypertension (≥140 mmHg systolic and/or ≥90 mmHg diastolic).

^b^Does not need to be defined proteinuria – any protein in the urine is sufficient.

^*^The presence of at least one of these clinical criteria for suspicion of preeclampsia is required for inclusion in the study.

#### Exclusion criteria

Women with manifest preeclampsia are excluded from the study. Manifest preeclampsia is defined as the presence of proteinuria ≥2+ by dipstick urinalysis (or ≥0.3 g protein/24 hours or ≥30 mg/dL protein in spot urine or spot urine protein/creatinine ratio ≥30 mg protein/mmol creatinine) AND reproducible elevated blood pressure (≥140 mmHg systolic and/or ≥90 mmHg diastolic) or current antihypertensive treatment. Women with a confirmed diagnosis of HELLP syndrome are also not eligible for study participation.

Other exclusion criteria include concomitant participation in another clinical study (with the exception of existing biobanks as agreed upon between individual clinical sites and the study sponsor); treatment with an investigational medicinal product during the 90 days prior to enrollment; being an employee at the investigational site; or being a relative or spouse of the investigator.

#### Definitions of preeclampsia-associated conditions and of maternal and fetal outcomes (diagnostic criteria)

To overcome any lack of consensus, specific diagnostic criteria for each preeclampsia-related disorder are defined in the protocol and must be strictly followed by all investigators: preeclampsia/eclampsia is to be diagnosed according to the criteria defined by the International Society for the Study of Hypertension in Pregnancy (ISSHP) and the American College of Obstetricians and Gynecologists (ACOG) guidelines [1, 20], with other established sources used to define specific criteria for HELLP syndrome [21], early-/late-onset preeclampsia [22], preterm delivery [23] and adverse neonatal outcomes (IUGR; small for gestational age) [24] (Table 2).

**Table 2:** **Definitions of preeclampsia-associated conditions and of maternal and fetal outcomes**

Condition/outcome	Definition
**Preeclampsia-associated conditions**	
Hypertension	• Systolic BP ≥140 mmHg and/or diastolic BP ≥90 mmHg (on two occasions ≥6 hours apart, but within 1 week)
	• Hypertension according to diagnostic criteria above (documented in medical history) controlled by antihypertensive drug use irrespective of current systolic and diastolic BP values
Chronic hypertension	• Hypertension (systolic BP ≥140 mmHg and/or diastolic BP ≥90 mmHg) diagnosed before conception or in the first half of pregnancy (<20 weeks of gestation) persisting >12 weeks postpartum
Proteinuria	• ≥0.3 g protein/24 hours
	• In emergency cases only if a 24-hour urine protein collection cannot be obtained: dipstick ≥2+ or ≥30 mg/dL protein in spot urine or spot urine protein/creatinine ratio ≥30 mg protein/mmol creatinine
Gestational hypertension	• New onset of hypertension (systolic BP ≥140 mmHg and/or diastolic BP ≥90 mmHg) alone without proteinuria after gestational week 20
Preeclampsia [1]	• New onset of both hypertension (systolic BP ≥140 mmHg and/or diastolic BP ≥90 mmHg) and proteinuria after 20 weeks’ gestation
Suspected preeclampsia	• Suspicion of clinical diagnosis of preeclampsia according to inclusion criteria
Severe preeclampsia [20]	Preeclampsia plus one or more of the following criteria:
	• Systolic BP ≥160 mmHg and/or diastolic BP ≥110 mmHg (on two occasions ≥6 hours apart, but within 1 week)
	• Proteinuria (>5 g protein/24 hours or dipstick ≥3+ on two random urine samples collected at least 4 hours apart)
	• Impaired renal function (serum creatinine ≥0.9 mg/dL or oliguria <500 mL/24 hours)
	• Pulmonary edema
	• Impaired liver function (elevated liver enzymes, epigastric or right upper-quadrant pain)
	• Neurologic symptoms (cerebral or visual disturbances, severe headache)
	• Hematologic disorders (thrombocytopenia, hemolysis)
	• IUGR
Eclampsia [20]	• New onset of tonic-clonic seizures in a woman with preeclampsia, which cannot be assigned to any other cause
Superimposed preeclampsia	• Chronic hypertension plus new onset of proteinuria after gestational week 20 or
	• Chronic hypertension and proteinuria before gestational week 20
	AND
	• Sudden increase of proteinuria or
	• Sudden increase of BP or
	• Clinical or laboratory signs/symptoms of severe preeclampsia
Early-/late-onset preeclampsia [22]	• Early-onset preeclampsia: onset at <34 + 0 weeks of gestation
	• Late-onset preeclampsia: onset at ≥34 + 0 weeks of gestation
HELLP syndrome [21]	• Increased aspartate transaminase (>70 IU/L)
	• Reduced thrombocyte counts (<100,000/μL)
	• Increased lactate dehydrogenase levels (>600 IU/L)
**Maternal and fetal outcomes**	
Intrauterine growth restriction [24]	• Estimated fetal weight or abdominal circumference <5th percentile (adjusted for gender and ethnicity according to the charts routinely used by the study site)
	• Presence of pathologic process that inhibits expression of normal intrinsic growth potential. Pathologic process to be demonstrated on at least one occasion after gestational week 22 by one of the below criteria:
	-Oligohydramnios (Amniotic Fluid Index <10th percentile)
	-Pathologic flow in umbilical artery (pulsatility index >95th percentile)
	• Serial ultrasonography growth curve anomalies*
	• Serial growth curve anomalies based on local measurement technique (manual measurement)*
Small for gestational age [24]	• Estimated fetal weight or abdominal circumference <5th percentile (adjusted for gender and ethnicity according to charts routinely used by the study site)
	• Absence of pathologic process (i.e. absence of pathologic criteria for oligohydramnios and umbilical artery flow as per IUGR criteria)
Preterm delivery [23]	• Birth before the completion of 37 weeks’ gestation (e.g. 36 weeks + 6 days is recorded as 36 completed weeks of gestation, so the baby is defined as preterm)

BP = blood pressure.

*Serial growth curve anomalies (measurement of symphysio-fundal height and serial ultrasound to determine divergence of head and abdominal circumference [25]) are used in UK sites only. Serial growth curve anomalies were used for suspicion of IUGR only, and diagnosis of IUGR had to be confirmed at delivery.

### Study procedures: assessments and data collection

**Figure 1:**
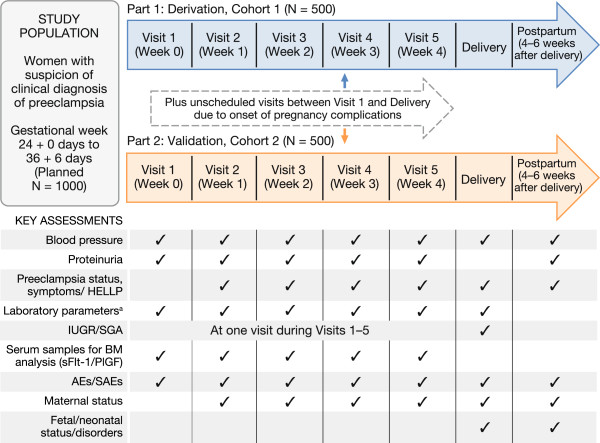
**Study design and key assessments.** Study design and data collection overview. AE = adverse event; BM = biomarker; HELLP = hemolysis, elevated liver enzymes and low platelets; IUGR = intrauterine growth restriction; PlGF = placental growth factor; SAE = serious adverse event; sFlt-1 = soluble fms-like-tyrosine kinase 1; SGA = small for gestational age. ^a^Laboratory parameters tested include thrombocyte counts and serum levels of aspartate aminotransferase, lactate dehydrogenase, and creatinine.

The study design and assessment plan is summarized in Figure 1. Part 1 of the study will use data from the first 500 eligible subjects enrolled to derive the sFlt-1/PlGF ratio cutoff-based short-term prediction model. The derived model will then be validated using data from a minimum of 500 additional subjects (part 2). Both parts of the study follow the same planned protocol schedule consisting of a baseline visit followed by four consecutive weekly visits prior to delivery, plus additional unscheduled visits in the event of preeclampsia-related pregnancy complications. A further study visit is scheduled at delivery, followed by a visit 4–6 weeks postpartum.

Clinical assessments, sampling for laboratory assessments and maternal sFlt-1/PlGF determination, and documentation of maternal and neonatal outcomes are to be performed at specified visits, as shown in Figure 1. Gestational age is calculated based on ‘last menstrual period’ or ‘first sonography date’ and recorded at visit 1. In addition, demographic data, medical history (including history of preeclampsia-related conditions and outcomes in previous pregnancies), body weight and routine pregnancy observations are documented at the baseline visit. Details of concomitant medication taken and occurrence of adverse events and serious adverse events are documented at each visit. Serious adverse events reported during the study are to be reported to the respective responsible authorities and the Ethics Committee/Institutional Review Board, according to applicable local regulatory requirements.

At each assessment, the preeclampsia status and its date of diagnosis are recorded: no preeclampsia, suspected preeclampsia (except at delivery and postpartum assessment), preeclampsia, severe preeclampsia, superimposed preeclampsia (a sudden increase in proteinuria or blood pressure in subjects with existing proteinuria and/or chronic hypertension before 20 weeks’ gestation), eclampsia or HELLP syndrome. The recognized categories of preeclampsia (particularly severe preeclampsia) have been updated since protocol development but, for consistency, site investigators will use the predefined criteria for inclusion of subjects in the analysis. These changes will not affect the primary analysis, which includes all cases of preeclampsia (severe and non-severe preeclampsia), but may need to be factored into secondary exploratory analyses where relevant.

The following preeclamptic symptoms are also documented: neurologic symptoms (headache, visual disturbances), epigastric pain, severe edema and oliguria.

#### Measurement of sFlt-1 and PlGF in maternal serum

Serum samples (minimum 2 mL) are collected at each study center in standard primary tubes according to a common standard operating procedure and sent to an independent laboratory for analysis. Single measurements are performed for sFlt-1 and PlGF on the fully automated Elecsys® system, as described previously [14, 18, 19] according to the procedure described in the respective product package inserts [26, 27]. The package inserts also report the precision that is achieved when the analytical test procedures are strictly followed [26, 27]. The sFlt-1/PlGF ratio is calculated for each sample.

#### Setting/locations

The study started in December 2010; during the first year, participants were enrolled at nine sites in Europe (Austria, Belgium, Germany, the Netherlands, Norway, Spain, Sweden [two sites] and the UK). In March 2012, the study was expanded to enroll participants at sites in Australia (five sites), Canada (four sites), New Zealand (two sites), Argentina (two sites), Chile (three sites), Peru (four sites) and an additional site in Germany. As of August 2013, 1273 subjects have been enrolled at 31 sites globally to ensure the eligible evaluable subject sample size was met, with the majority of participants in European centers. Enrollment is now complete.

### Statistical methods

Analysis is to be conducted in two parts: an interim analysis corresponding to the derivation part of the study, and the main analysis corresponding to the validation of the derived cutoff algorithms. The study is powered for the primary objectives, for which the interim analysis requires 500 eligible participants. A separate similarly sized cohort is required to validate the model.

#### Sample size calculations

Sample size calculations were performed according to Pepe [28] and based on the anticipated performance of Elecsys® sFlt-1/PlGF as derived from a previous study [14] (assuming a sensitivity of 95% and false positive rate of 15%), suggestions by medical experts on minimal requirements for the positive prediction value (PPV) and negative predictive value (NPV) and an assumed prevalence of 15% in a population of women with signs and symptoms of preeclampsia (conservative estimate based on a prevalence of preeclampsia of 3–5% in the whole population [2]). Based on these values, a sample size of approximately 1000 was set, with an interim analysis deemed appropriate after recruitment of 500 subjects because of the uncertainty about the preeclampsia prevalence in the target population.

The purpose of the interim analysis was to derive a valid diagnostic algorithm if the data set proved to be sufficient to do so, or to continue sampling until the appropriate sample size had been reached. Depending on the test performance results in the derivation part of the study, the sample size of subjects for the validation study would be adjusted accordingly.

At the time of writing, the first 500 participants have completed the study; preeclampsia prevalence in the first 500 eligible participants is approximately 20% [29]. The sample size for the validation study has been adjusted based on estimates for NPV and PPV as well as the prevalence of positive values (above the cutoff at baseline) as obtained in the derivation part. As a consequence, 500 subjects in the validation part should provide sufficient numbers of negative as well as positive values to simultaneously confirm the estimates for NPV and PPV as obtained in the derivation part (α = 5%; power = 90%).

#### Primary analyses

Three main groups of subjects are defined for the primary analyses:

-  Group 1: Subjects who develop preeclampsia/eclampsia/HELLP syndrome within the first week after the baseline visit.
-  Group 2: Subjects who develop preeclampsia/eclampsia/HELLP syndrome after the first week and within 4 weeks after the baseline visit.
-  Group 3: Subjects who do not develop preeclampsia/eclampsia/HELLP syndrome within 4 weeks after the baseline visit.

In the statistical model for prediction of absence of diagnosis of preeclampsia/eclampsia/HELLP syndrome within 1 week, Group 1 will be compared with Groups 2 and 3. In the model for predicting occurrence of preeclampsia/eclampsia/HELLP syndrome within 4 weeks, Groups 1 and 2 will be compared with Group 3. Prediction performance of the sFlt-1/PlGF ratio is defined by an NPV in the first model and a PPV in the second model.

### Part 1: Derivation study

Short-term prediction algorithms will be derived for both outcomes based on sFlt-1/PlGF cutoffs and – if found to enhance prediction performance of the model – gestational age. Therefore, six different prediction models will be calculated. Three model types are applied to each of the two prediction items, rule-out for 1 week and rule-in within 4 weeks: one model using a single cutoff (independent of gestational age); one model using two cutoffs – one for early gestational phase (24 weeks + 0 days to 33 weeks + 6 days), one for late gestational phase (34 weeks + 0 days and later); and one model using cutoffs for each week of gestation. Estimates for NPV, PPV, sensitivity and specificity representing the performance of each of these models are calculated using stratified Monte Carlo cross-validation with 1999 replicates and a training-test-ratio of 2:1 [30, 31].

### Part 2: Validation study

One of these model types (for each of the two prediction items, rule-out for 1 week and rule-in within 4 weeks) is to be selected for the validation part, based on prognostic performance in terms of NPV and PPV determined in the derivation study, combined with feasibility for practical clinical utility. Prediction performance will be reported by estimates of NPV, PPV, clinical sensitivity and specificity, area under the curve (AUC) with receiver operating characteristics (ROC) curves, with corresponding 95% confidence intervals (CIs).

#### Analysis populations

Data from available studies suggest that the serum levels of sFlt-1 and PlGF are different in women with multi-fetal pregnancies [32, 33]. Therefore, only women with singleton pregnancies will be included in the primary objective analysis. Subjects included in the primary analyses must have sufficient data available from scheduled and unscheduled visits to determine absence or occurrence of preeclampsia/eclampsia/HELLP syndrome within 1 and 4 weeks of the baseline visit.

#### Secondary analyses

Analysis of the sFlt-1/PlGF ratios in women with versus without preeclampsia-related adverse outcomes will be described by descriptive statistics and illustrated by box-plot graphs. The ROC curve separating these groups will be shown with the according AUC value plus individual 95% CI.

A nested case-control analysis will be performed to evaluate the use of sFlt-1, PlGF and the sFlt-1/PlGF ratio as an aid in diagnosis. The ROC curve separating women with versus without preeclampsia/eclampsia/HELLP syndrome will be shown with the corresponding AUC estimates for the sFlt-1/PlGF ratio as well as for the single-marker values of sFlt-1 and PlGF for the whole gestational phase and for both early and late gestational phases separately.

Weekly increases in sFlt-1/PlGF ratios will be represented by slopes of the sFlt-1/PlGF ratio values between consecutive visits, adjusted to reflect an exact 7-day interval. Slope values in subjects with versus without preeclampsia/eclampsia/HELLP syndrome within 4 weeks after the baseline visit will be described by descriptive statistics and illustrated by box-plot graphs. Logistic mixed-effects models will be applied.

To assess correlation between preeclampsia severity and the sFlt-1/PlGF ratio, analyses based on weekly slope values (prior to diagnosis of preeclampsia) and sFlt-1/PlGF ratios (in diagnosed cases) will be applied to subgroups of subjects with preeclampsia, superimposed preeclampsia or severe preeclampsia/eclampsia/HELLP syndrome.

sFlt-1/PlGF ratios from participants with or without preterm delivery (<37 weeks of gestation) and with or without development of preeclampsia/eclampsia/HELLP syndrome will be described in parallel by descriptive statistics.

Association between time to delivery and the sFlt-1/PlGF ratio will be assessed by appropriate regression methods such as accelerated failure time (e.g. using Weibull or log-logistic distribution) or proportional hazard (e.g. Cox) models.

#### Health economics analysis

Hospitalization and outcomes data for the mother and neonate will be used to build a health economics model to evaluate the cost-effectiveness of adding the sFlt-1/PIGF ratio test to standard practice for short-term prediction of preeclampsia as a secondary analysis.

### Ethics statement

Each participating study site provided Ethics Committee/Institutional Review Board approval of the study protocol and associated documents (participant informed consent, participant information) before the start of the clinical part of the study. All women provided written informed consent before enrollment.

## Discussion

Reliable prediction of preeclampsia and related clinical maternal and fetal adverse outcomes is a high unmet medical need in pregnancy care. In current clinical practice, diagnosis of preeclampsia relies largely on the measurement of blood pressure and proteinuria [1, 20], even though both measures are poorly predictive of adverse maternal and fetal outcomes and complications. Due to the unpredictable nature and potential severity of adverse outcomes associated with preeclampsia, women with suspected preeclampsia may need to be hospitalized for close observation and monitoring (frequent laboratory testing and evaluation of fetal wellbeing) [1, 20, 34]. However, some women with diagnosed preeclampsia carry pregnancy almost to full term without complications [35]. The ability to rule out the likelihood of preeclampsia developing in at-risk women through a negative predictive test would be an important advance in pregnancy care and would enable valuable resources to be directed to the patients who need them most.

The sFlt-1/PlGF ratio is a valuable aid in the diagnosis of preeclampsia and discriminates between different types of pregnancy-related hypertensive disorders [15]. The most recent data from a case-control study defined separate cutoff values for early-onset preeclampsia and late-onset preeclampsia [17]. In women with suspected preeclampsia, the sFlt-1/PlGF ratio may also help to identify those women who will develop a preeclampsia-related pregnancy complication [35].

PROGNOSIS is the first clinical study evaluating short-term prediction of preeclampsia using fully automated Elecsys® sFlt-1/PlGF maternal blood testing in pregnant women with clinical suspicion of preeclampsia. Studies such as the 3500+ patient Screening for Pregnancy Endpoints (SCOPE) study have recruited women on the basis of risk factors associated with preeclampsia (e.g. first pregnancy) [36]. In SCOPE, PlGF failed to achieve clinical utility as a screening factor alongside established clinical risk factors for developing preeclampsia later in pregnancy. PROGNOSIS is designed to recruit subjects with at least one clinical sign of preeclampsia (i.e. elevated blood pressure or protein in the urine, or other preeclampsia-related symptoms), and therefore at higher risk of developing preeclampsia.

PROGNOSIS is the largest study to date in which the predictive performance of angiogenic serum markers is evaluated in women with suspicion of preeclampsia based on clinical signs and symptoms. Since recruitment started in December 2010, 1273 participants have been enrolled, and recruitment is now complete. Smaller target populations have been recruited in other studies evaluating the predictive performance of angiogenic markers in preeclampsia. In a prospective, observational UK cohort study of 625 women with suspected preeclampsia (20–40 weeks of gestation), a low PlGF level was highly sensitive as a negative predictive marker for determining which women will require delivery within 14 days, although the findings remain to be validated [37]. In the US, a prospective cohort study of 85 women with suspected preeclampsia at 20–36 weeks’ gestation showed that addition of the PlGF/sVEGFR-1 ratio to standard clinical tests improved identification of women who required delivery due to preeclampsia within 2 weeks [38].

Eligible participants in our study were selected using protocol-defined clinical criteria to ensure consistency across study centers. Specific diagnostic criteria for each hypertension- and preeclampsia-related condition/outcome, were also defined (sourced from appropriate guidelines [1, 20–24]). Overall, there was good compliance with the defined diagnostic criteria with source data verification performed for all sites; this ensured that clinical diagnosis of preeclampsia was largely consistent between centers. Strict adherence to a uniform set of diagnostic criteria in a global setting will also give valuable information regarding the real prevalence of preeclampsia/eclampsia/HELLP syndrome in a high-risk population.

Another important feature of PROGNOSIS is the double-blind design, which ensures that the investigator’s knowledge of an individual participant’s sFlt-1/PlGF status has no influence on the diagnosis of preeclampsia or related conditions, and has no impact on clinical treatment decisions.

There is increasing recognition that preeclampsia is a systemic multi-organ condition for which clinical criteria alone are inadequate to predict adverse outcomes. The potential utility of angiogenesis-related biomarkers, including the sFlt-1/PlGF ratio, for predicting preeclampsia are acknowledged in the latest guideline updates from DGGG (German Society of Obstetrics and Gynecology) [39] and ACOG [40]. PROGNOSIS will provide the most comprehensive evidence to date on the accuracy of the sFlt-1/PlGF ratio as a short-term predictive marker for preeclampsia. Accurate prediction of preeclampsia has the potential to reduce the frequency of adverse maternal and fetal outcomes, including iatrogenic preterm delivery, and to decrease healthcare costs associated with hospitalization.

## Abbreviations

ACOG: American College of Obstetricians and Gynecologists
AE: Adverse event
AUC: Area under the curve
BM: Biomarker
BP: Blood pressure
CE: Conformité Européenne
CI: Confidence interval
HELLP: Hemolysis, elevated liver enzymes and low platelets
IUGR: Intrauterine growth restriction
ISSHP: International Society for the Study of Hypertension in Pregnancy
IVD: In vitro diagnostic
NPV: Negative predictive value
PlGF: Placental growth factor
PPV: Positive predictive value
PROGNOSIS: PRediction of short-term Outcome in preGNant wOmen with Suspected preeclampsIa Study
ROC: Receiver operating characteristics
SAE: Serious adverse event
SCOPE: Screening for Pregnancy Endpoints
sFlt-1: Soluble fms-like-tyrosine kinase 1
SGA: Small for gestational age
sVEGFR-1: Soluble vascular endothelial growth factor receptor 1
VEGF: Vascular endothelial growth factor.
